# The prevalence of HTLV-1 and its Co-Infection with HCV, HBV and HIV in Hemophilic patients

**DOI:** 10.12669/pjms.315.7888

**Published:** 2015

**Authors:** Masood Ziaee, Mohammad Hassan Namaei, Ghodseh Azarkar

**Affiliations:** 1 Masood Ziaee, Hepatitis Research Center, Birjand University of Medical Sciences, Birjand, Iran; 2 Mohammad Hassan Namaei, Hepatitis Research Center, Birjand University of Medical Sciences, Birjand, Iran; 3 Ghodseh Azarkar, Hepatitis Research Center, Birjand University of Medical Sciences, Birjand, Iran

**Keywords:** Hemophilia, Transfusion-Transmitted Virus, Hepatitis C virus, Hepatitis B Virus, HTLV-1

## Abstract

**Background and Objective:**

Blood-borne infections, such as the HIV virus and hepatitis B and C, are major problems in patients receiving blood products. Here we examined the prevalence of HTLV-1, HCV, HBV, and HIV in hemophilic patients.

**Methods:**

A cross-sectional study on 108 hemophilic patients (101 males and 7 females) involved detection of HBV, HCV, HIV and HTLV-1 infections using immunoassays for HBsAg, hepatitis B core antibodies (anti-HBc), hepatitis C antibodies (anti-HCV), HIV antibodies (anti-HIV) and Anti-HTLV-1. Real-time PCR was used to measure HCV RNA, and HCV genotyping was performed by direct sequencing of the 5’ noncoding region.

**Results:**

Hemophilia A was reported in 93 (86%) patients with severe symptoms in 8 cases. The seroprevalence of anti-HCV and anti-HTLV-1 antibodies was 20% and 3% respectively. One patient with severe hemophilia had a HCV/HTLV-1 co-infection. HCV-RNA was detected in 82% of patients. In terms of genotyping prevalence was 56% HCV genotype 3a, 39% HCV genotype 1a, and 6% HCV genotype2. Anti HIV and HBsAg were not detected in any patient. HTLV1 prevalence was higher, HCV lower in South Khorasan than other regions in Iran or elsewhere.

**Conclusion:**

Management of transfusion of blood and blood products should account for the underlying prevalence of infectious agents.

## INTRODUCTION

Hemophilia is a disease characterized by serious bleeding symptoms due to a deficiency of factor VIII for hemophilia A and factor IX for hemophilia B (Christmas disease).^1^ Bleeding episodes were previously treated with whole blood transfusions. The management of hemophilia has dramatically improved in the last 25 years.^2^ However, these therapies based on human properties have always been associated with the risk of microbiological transmission, especially viral infections such as human immunodeficiency virus (HIV), hepatitis C virus (HCV), and hepatitis B virus (HBV). A potential risk of virus transmission by these blood products persists because the serological screening of blood donors cannot detect all potential infections. As a result, screening blood donors for HIV-1 and HCV RNA by means of nucleic acid amplification was introduced in 1999 in developed countries to identify donations made during the window period before seroconversion.^3^ Besides, patients with hemophilia remain at risk of contamination by non-inactivated products such as cryoprecipitate and/or nosocomial infections. Specifically, much attention has been drawn to the safety issues of handling blood and blood products after the worldwide accidental viral contamination among hemophilia patients.^4^

According to the global survey carried out by the World Federation of Hemophillia, Iran ranked second in the eastern Mediterranean region after Egypt having the largest number of hemophilia cases. Currently in Iran, there are about 7000 hemophilia patients^5^ and all have access to commercial concentrated coagulation factors free of charge. The prevalence of HIV in Iranian hemophilia patients was reported between 0% and 5%.^6^-^8^ Also, the rate of HCV infection was reported from about 22% to 66% among hemophilic patients in Iran.^8^-^10^ Despite the relatively low incidence of viral infection among hemophilia patients in Iran compared to other developed countries, about 3000 hemophiliacs (42%) have filed lawsuits against the Iranian national health care system since the 1990s claiming compensation for their infection.^5^ Thus, the purpose of the current study was to investigate the seroepidemiology of these infections such as HTLV1 and other main pathogens transmitted by blood and blood products.

## METHODS

According to the Southern Khorasan branch of Iranian Hemophilia Society in Birjand, there were 108 officially registered patients between 2010 and 2012. This cross-sectional study was conducted on all these 108 hemophilic patients in the Sothern Khorasan province. The protocol of the study was approved by the Ethics Committee of Birjand University of Medical Sciences, Birjand, Iran. The subjects were given a clear explanation of the objectives of the study as well as the potential risks involved. Consent forms (parental consent forms for those below 18 years of age) were obtained for all subjects.^11^ Five ml of venous blood was collected from each patient and the serum was placed in sterile covered storage tubes and stored at −30°C until all samples were collected.^12^

All serum samples were tested by commercially available enzyme-linked immunosorbent assay (ELISA) kits to detect hepatitis B surface antigen (HBsAg; Enzygenost® HBS Ag 5.0, Dade Behring Inc. Newark USA), hepatitis B core antibody (anti-HBc; bioELISA anti HBc, Biokit, Barcelona, Spain), Anti-hepatitis C Virus (anti-HCV; Hepanostika HCV ultra, Beijing United Biomedical Co. LTD. Beijing, China), Anti-HIV antibodies (HIVAb; Genscreen® plus HIV Ag-Ab, Bio-Rad, Matnes la coquette, France), and human T-cell leukemia virus antibodies (HTLV-1 Ab; Gene labs® Diagnostics HTLV-I/II Elisa 3.0 Geneva, Switzerland). All sera positive for HCVAb were retested by the second generation of recombinant immunoblot assay (RIBA) kits (HCV blot 3.0; Genelabs Diagnostics®, Singapore) as a complementary test. Serum HCV-RNA was detected by a nested reverse transcription RT-PCR and HCV genotyping was performed by direct sequencing of the 5’ noncoding region. The severity of hemophilia was based on plasma procoagulant levels, with persons < 1% factor defined as severe; 1 to 5% as moderate; and > 5% as mild.^13^

Descriptive statistics were performed using the SPSS statistical software (SPSS, Chicago, Illinois, USA, version 19). Data are presented as mean ± SD and range for quantitative variables. Categorical data are presented as numbers and percentages.

## RESULTS

The mean age of participants was 27.7 years (±16.4 years) with a range of four to 85 years. The majority (93.5%) of the patients were males (*N* = 101). The hemophilia A was reported in 93 (86.1%) patients, and the severity of disease was mostly (87.6%) mild (*N* = 92). The most common blood groups were, in descending order, group B-positive (38.0 %) and A-positive (25.9%). Descriptive statistics of the patents are presented in Table-I.

**Table-I:** Demographic and descriptive disease statistics of hemophilic patients (*N* =108).

Variables	Frequency	Percentage
Age (mean±SD)	27.7 years ±16.4	Range: 4-85 years
***Gender***		
Male	101	93.5%
Female	7	6.5%
***Marital status***		
Single	51	47.2%
Married	57	52.8%
***Blood group***		
B+	41	38.0%
A+	28	25.9%
O+	22	20.4%
AB+	9	8.3%
O-	5	4.6%
A-	2	1.9%
AB-	1	0.9%
***Type of disease***		
Hemophilia A	93	86.1%
Hemophilia B	2	1.9%
Von Willebrand disease	4	3.7%
Platelet dysfunction	1	0.9%
Other	8	7.4%
***Severity of disease***		
Mild	92	87.6%
Moderate	5	4.8%
Severe	8	7.6%

**Fig.1:**
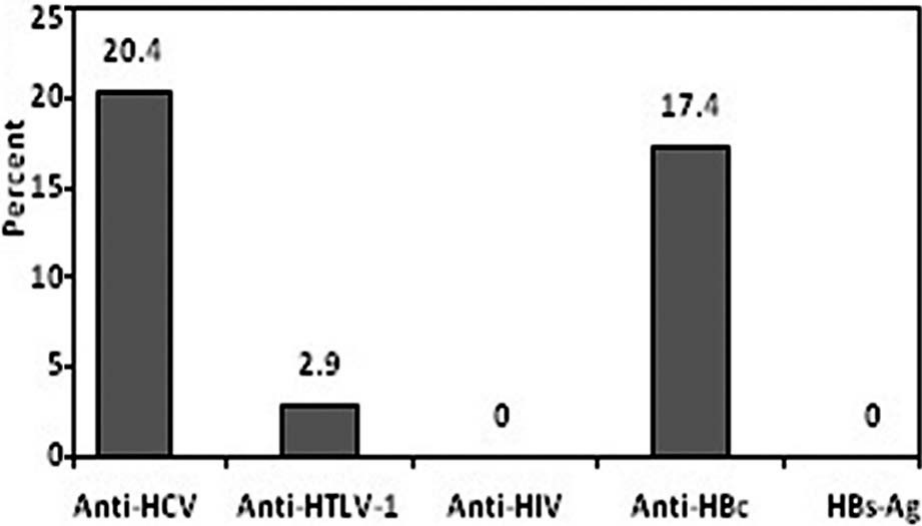
Results of HBV, HCV, HIV, and HTLV-1 serological test among hemophilic patients.

The seroprevalence of anti-HCV antibody was 20.4% (22 of the 108 patients); whereas 18 (82%) of these 22 patients who tested positive with anti-HCV also had positive results in the RT-PCR method. Among these 18 positive results, HCV genotype was determined and there were 10 (55.5%) patients infected with genotype 3a, seven patients (39%) with genotype 1a, and one patient (5.5%) with genotype 2a. Three cases (2.9%) had a positive anti-HTLV-1 serology. One of the patients with severe hemophilia had a HCV/HTLV-1 co-infection. All patients with positive anti-HCV or anti-HTLV-1 antibody were born before 1994. All patients were negative for HIV antigen and HBsAg as well. As depicted in Figure 1, a total of 15 (17.4%) patients were tested positive for antibody to anti-HBc. All those 18 patients with a positive HCV RNA were treated with interferon alpha plus ribavirin and had a sustained viral response to treatment at follow-up.

## DISCUSSION

According to sequencing of 5’ noncoding region in HCV positive samples, there were 10 (55.5%) patients infected with genotype 3a, 7 (39%) with genotype 1a and 1 (5.5%) with genotype 2a. Other researches revealed that genotype 1a, 3a and 1b are the most common genotypes in Iran.^14^ In this study, there was neither any case of HCV with genotype 1b nor mixed genotype. This is probably due to the sample size and the genotyping method being used. The 5’ noncoding region in HCV genome is much conserved among genotypes and is also very common to distinguish genotypes, although it may be missed some subtypes in direct sequencing of 5’ noncoding. Many researchers have studied the rate of viral infection in hemophilic patients in Iran and worldwide. The reported prevalence of HCV infection is different in various areas. In Iran, a study on 236 hemophilic patients reported a high rate of 83.3% HCV seropositive in Tehran.^6^

Other reports from Isfahan and Ahvaz on hemophilic patients demonstrated that the prevalence of HCV infection was 66% and 54%, respectively.^9^,^10^ Also, some lower rates of HCV infection among hemophilic patients were described in Iran, such as 22.6% (between 1993 and 2006 in Isfahan) in a study by Shakeri et al.^15^ and 29.6% (between 2003 and 2006 in Zahedan) in a study by Sharifi-Mood et al.^8^ which are closer to our results. In the current study, however, the rates of HCV were lower in young subjects and did not observed in patients who were born after 1994. Prevalence found in Iranian hemophiliacs seems to be greatly higher than those reported in the general population.^15^,^16^ This indicates an increased risk of contamination evident among hemophilia patients. Data from the Iranian literature in the general population reported prevalence of 0.16% (95% confidence interval [CI]: 0%-0.59%) for HCV infection.^17^ Also, the prevalence of HTLV-1 infection among general population in three studies in Khorasan province, a known endemic region for HTLV-1 infection compared to other regions in the world, was reported 1.66%, 2.1%, and 7.2% in Sabzevar, Mashhad, and Neyshabour cities, respectively.^18^-^20^

Furthermore, in a previous study on hemophilic patients in Sothern Khorasan province, 1.25% of patients had a HTLV-1 infection.^21^ It is much more important in hemophilia born before 1994, the date of introduction of the inactivation processes of coagulation factor concentrates. In Iran, hemophiliacs were treated with blood products without viral inactivation treatment before 1997^6^. A cumulative effect may be due to the risk of infection by non-inactivated cryoprecipitate. HCV infection is even lower in hemophiliacs for those born after 1990s, the date of introduction of routine screening for HCV in blood donation in Iran. Thus, the potential risk of infection has become very unlikely after the 1990s.

Although all of our patients had no positive HIV infection, international literature has reported high frequencies (15-45%) of HIV infection in hemophiliacs before almost simultaneous introduction of the inactivation processes and screening of anti-HIV among blood donors.^22^,^23^ The rate of HIV infection has declined further, due to deaths and an increasing number of hemophilic patients born after plasma products became safe.^24^ However, other Iranian studies indicated a range from 0% to 5% for HIV infection among hemophiliacs.^6^-^8^

### Ethical Approval

The protocol of the study was approved by the Ethics Committee of Birjand University of Medical Sciences, Birjand, Iran, also all procedures performed in this study were in accordance with the ethical standards of the 1964 Helsinki declaration and its later amendments or comparable ethical standards. The subjects were given a clear explanation of the objectives of the study as well as the potential risks involved. Consent forms (parental consent forms for those below 18 years of age) were obtained for all subjects.
